# Nipah Virus: Past Outbreaks and Future Containment

**DOI:** 10.3390/v12040465

**Published:** 2020-04-20

**Authors:** Vinod Soman Pillai, Gayathri Krishna, Mohanan Valiya Veettil

**Affiliations:** Virology Laboratory, Department of Biotechnology, Cochin University of Science and Technology, Cochin 682022, Kerala, India; vinodsoman08@gmail.com (V.S.P.); gayathriradhan@gmail.com (G.K.)

**Keywords:** emerging virus, Nipah, outbreak, transmission, prevention, control

## Abstract

Viral outbreaks of varying frequencies and severities have caused panic and havoc across the globe throughout history. Influenza, small pox, measles, and yellow fever reverberated for centuries, causing huge burden for economies. The twenty-first century witnessed the most pathogenic and contagious virus outbreaks of zoonotic origin including severe acute respiratory syndrome coronavirus (SARS-CoV), Ebola virus, Middle East respiratory syndrome coronavirus (MERS-CoV) and Nipah virus. Nipah is considered one of the world’s deadliest viruses with the heaviest mortality rates in some instances. It is known to cause encephalitis, with cases of acute respiratory distress turning fatal. Various factors contribute to the onset and spread of the virus. All through the infected zone, various strategies to tackle and enhance the surveillance and awareness with greater emphasis on personal hygiene has been formulated. This review discusses the recent outbreaks of Nipah virus in Malaysia, Bangladesh and India, the routes of transmission, prevention and control measures employed along with possible reasons behind the outbreaks, and the precautionary measures to be ensured by private–public undertakings to contain and ensure a lower incidence in the future.

## 1. Introduction

Nipah virus (NiV), a bat borne pathogen, that causes lethal encephalitis in humans has recently been reported from Malaysia, Bangladesh, Singapore and India [1,2,3]. It is one among the emerging deadly zoonotic viruses and is included in the order Mononegavirales among other dreaded viruses such as Hendra, Ebola and Marburg [4]. Pteropus fruit bats are considered the natural reservoirs of the virus [5,6]. NiV was discovered in 1998 during the first reported outbreak in the Sungai Nipah, a village in Malaysia, where humans contracted NiV from pigs, the intermediate hosts of the virus [7,8]. Recurring NiV outbreaks have been reported annually in different parts of Bangladesh from 2001, where the infection occurred due to the consumption of raw date palm sap contaminated with saliva and excreta of the bats. In India, the first outbreak occurred in Siliguri, West Bengal in 2001 with mostly nosocomial or person-to-person close contact, and in 2007, a repeated outbreak was reported in Nadia in West Bengal [7,9]. Recently, in 2018, a NiV outbreak was recorded in the Kozhikode district of Kerala, a South Indian state where the index patient was reported to have contracted NiV from fruit-eating bats. However, no clinical or statistical evidence was available to prove the incidence, though the spread was mostly through nosocomial infection. All the outbreaks have recorded high rates of fatality including the 91% mortality rate during the recent Kerala outbreak [10].

Outbreaks of various zoonotic viruses occur as a result of the influence of several factors including human-to-human contact and animal interaction with severe environmental changes [11]. Ecological, environmental and anthropogenic factors might contribute intensively to the recent zoonotic outbreaks of NiV [12,13]. Anthropogenic factors have led to an emergence of viral diseases by creating a drastic imbalance in the ecosystem and the environment. Climate change, depleting resources, deforestation and changes in natural terrains, farming and industrialization are also significant factors which result in the outbreaks of viral diseases. In this context, we describe the epidemiological background of the earlier NiV outbreak, the mode of transmission, preventive and control measures and the factors that may have contributed to the outbreak. 

## 2. Nipah Virus (NiV)

**Figure 1:**
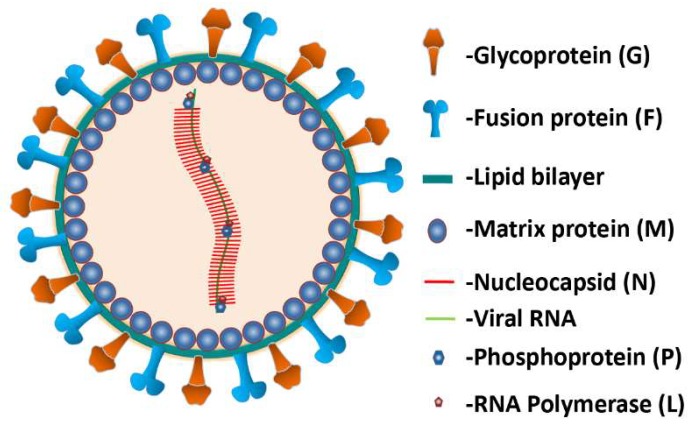
Representation of Nipah virus (NiV) structure. The RNA genome attached to the N, P, and L proteins form the ribonucleoprotein complex which is surrounded by a lipid bilayer envelope studded with two envelope glycoproteins G and F. The RNA encodes six structural proteins: N, P, M, F, G and L. The matrix protein M associates with the inner part of the envelope. The structural proteins, RNA and lipid bilayer are indicated in different colors.

NiV is an enveloped pleomorphic virus belonging to the genus Henipavirus of the family Paramyxoviridae [14]. The genome of the virus consists of a non-segmented negative sense single-stranded RNA of approximately 18.2 kb long which encodes six structural proteins: nucleocapsid (N), phosphoprotein (P), matrix protein (M), fusion protein (F), glycoprotein (G) and RNA polymerase (L) [15]. The N, P and L along with the viral RNA forms the ribonucleoprotein complex, an indispensable complex that regulates transcription and viral RNA synthesis (Figure 1). 

Attachment and entry into the host cell is controlled by the F and G proteins spanning across the envelope [16]. The G protein mediates virus attachment and binds to the host cellular Ephrin-B2 and -B3 receptors while the F protein induces viral-cell membrane fusion facilitating the entry of virion [17,18]. 

### 2.1. Epidemiology 

**Figure 2:**
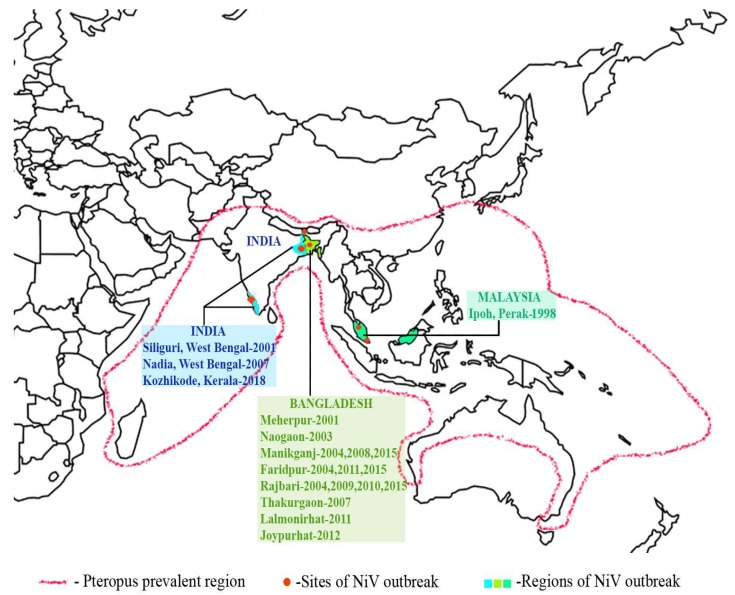
Map of NiV outbreaks and Pteropus fruit bats distribution. In the map, the sites of NiV outbreaks in India, Bangladesh and Malaysia are depicted in different colors. Pteropus bat (the major carriers of NiV) prevalent regions are demarcated by red-dotted line.

Fruit bats of the genus Pteropus (flying foxes) are the natural reservoirs for NiV [5,6]. They feed on fruits and nectar and are found predominantly in areas around farms and orchards, limiting the barrier of spillover of the viruses [5]. The bats are endemic to tropical and subtropical regions of Asia, East Africa, Australian continents and some oceanic islands and are proved to be associated with the NiV outbreaks reported in different parts of the world (Figure 2) [5,6]. Being the natural hosts for NiV, bats are symptomless carriers but they shed the viruses in their saliva, urine, semen and excreta [6,19]. A few fruit bat populations in Cambodia [20], Thailand [21], Madagascar [22] and Ghana [23] have been found positive for NiV-neutralizing antibodies during the serological surveillance studies. The route of transmission occurs via contact with excretions or secretions of infected animals, ingestion of fruit contaminated with NiV or close contact with infected human bodily fluids [24,25].

### 2.2. Transmission of NiV

In Malaysia, humans contracted the disease through NiV-infected pigs, the intermediate hosts of the virus [26]. Spillover of the virus from bats to pigs are due to the consumption of fruits partially eaten or contaminated by bats infected with NiV [27]. Transmission of the virus from pig to human occurred through direct contact with the infected pigs, and human-to-human infection could be through direct contact, aerosols or fomites. 

**Figure 3:**
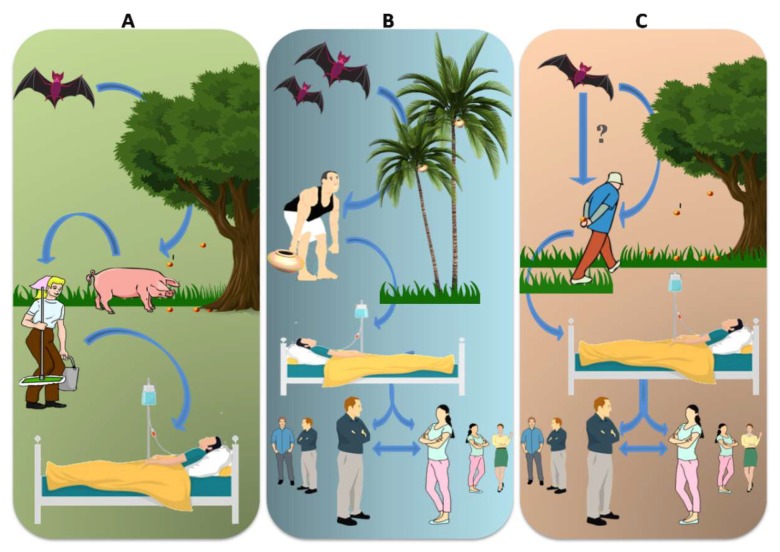
Routes of NiV transmission. Different locations have different routes of transmission. (**A**) In Malaysia, bat bitten fruits contaminated with NiV-M were consumed by pigs and workers handling the pigs were infected with NiV-M. (**B**) In Bangladesh, bat saliva- and excreta-contaminated palm sap consumption lead to NiV-B infection in humans and was spread further via nosocomial mode. Infected bats shed the virus in their urine, excreta and saliva. (**C**) In India, the possibility of direct bat-to-human transmission has been reported in Kerala state, but this was not supported by adequate evidence. Nosocomial spread of NiV-B have been reported in two different states—Kerala and West Bengal.

Abattoir workers are often in contact with excretions and secretions such as the urine, saliva, pharyngeal and respiratory secretions of infected pigs or raw pig meat and other products contaminated with NiV [28] (Figure 3A). As pigs developed severe respiratory discomfort, aerosol spread of NiV to humans is also considered an important respiratory route of transmission [29]. Importation of pigs from Malaysia to Singapore led to the spread of infection to the pig farmers living in close proximity to the pig sties [28]. 

Investigations in Bangladesh report that a few infected patients had consumed raw palm sap about 30 days prior to the disease onset, suggesting the contamination of palm sap with secretions of NiV-infected bats [9]. During the outbreak, a high rate of inter-person transmissions was also reported [30,31] (Figure 3B).

Outbreaks in Bangladesh and India (West Bengal and Kerala) share epidemiologic similarities concerning human-to-human transmission of NiV with no intermediate hosts [31]. The outbreaks were predominantly nosocomial and mostly affected hospital staff, caregivers and bystanders [32]. Outbreaks in Siliguri are assumed to have originated from bats. In Kerala, the index patient, the first infected individual of the community is believed to have contracted NiV from infected fruit bats, though this information is not substantiated (Figure 3C). All other patients contracted the disease through nosocomial transmission and hence person-to-person transmission frequency was high in Kerala and similar to the rates in West Bengal and Bangladesh [10,32].

### 2.3. Outbreaks

**Figure 4:**
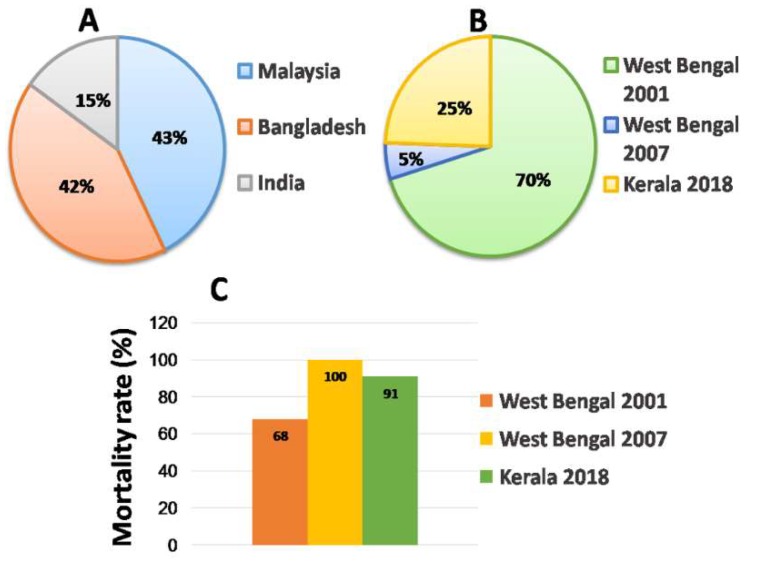
Outbreak statistics. (**A**) NiV outbreak across the world with percentage of outbreaks in Malaysia, Bangladesh and India individually. (**B**) State- and year-wise NiV outbreaks in percentage in India. (**C**) NiV mortality rate in the Indian states of West Bengal and Kerala.

NiV outbreaks have so far been reported in three countries: Malaysia, Bangladesh and India. Although there are surveillance studies proving the presence of antibodies from other nations including Cambodia [20], Thailand [21], Madagascar [22] and Ghana [23], Malaysian outbreaks represent 43% of NiV incident cases and Bangladesh and India represent 42% and 15% respectively of the total incident cases worldwide (Figure 4A). 

#### 2.3.1. Malaysia

It was in September 1998 when the first case of outbreak began among the farmers in the pig farming industry near the city of Ipoh in the state of Perak, Malaysia. The second outbreak occurred in the state of Negri Sembilan at the end of December 1998. During this period, a similar and third outbreak was reported in the nearby areas of Bukit Pelandok [8,33,34]. In the beginning, the causative agent for the outbreaks was mistaken to be Japanese encephalitis virus and hence preventive measures like immunizations for Japanese encephalitis virus and mosquito-controlling were undertaken to contain the outbreaks [33]. Despite all the control measures, new cases were still on the rise and many pigs died with severe respiratory difficulties which ruled out the possibility of a Japanese encephalitis virus infection [35]. By March 1999, scientists confirmed the presence of a novel virus, Nipah [36,37]. Close contact with NiV-infected pigs was recognized to be the source of infection ensuring transmission to humans. NiV was contained by May 1999, by then 105 deaths occurred from a total of 265 cases with 39.6% mortality [38].

Meanwhile NiV had spread to Singapore in late February 1999, following the importation of infected pigs from Malaysia, where 11 abattoir workers contracted the disease with one case of death [28]. Immediate action such as the culling of pigs and banning the import of pigs from Malaysia helped to contain the infection by May 1999 [26,39].

#### 2.3.2. Bangladesh

The first outbreak was recorded in the district of Meherpur, Bangladesh in April 2001 with 13 diagnosed cases [30]. Down the line, many outbreaks of NiV occurred annually at various parts of Bangladesh like Naogoan, Rajbari, Faridpur, Tangail, Thakurgaon, Kushtia, Pabna, Natore, Manikganj, Gaibandha, Rangpur, Nilphamari, Madaripur, Gopalganj, Lalmohirhat, Dinajpur, Comilla, Joypurhat, Rajshahi, Jhenaidah, Mymensingh, Ponchoghor and Magura from April 2001 to February 2015, out of which a few districts were affected with recurrent outbreaks [40]. Though transmission was predominantly through the consumption of NiV-contaminated raw date palm sap, poor surveillance and medical facilities augmented the mortality rates [9,41]. Approximately 261 confirmed cases with 199 deaths were reported with a mortality rate of 76.2% up until 2015 [42].

#### 2.3.3. India

The first outbreak in India was reported between January and February 2001 in the district of Siliguri, a leading commercial city of West Bengal. Because Siliguri borders Bangladesh and because of a failure of initial laboratory investigations to discover the causative agent, patient samples were retroactively tested for the NiV virus [2,43]. The serum samples of 9 patients out of 18 tested positive for NiV-specific immunoglobulin M (IgM) and immunoglobulin G (IgG) antibodies while NiV RNA was detected in 5 urine samples. This serious outbreak caused 45 deaths from a total of 66 confirmed patients with a mortality rate of 68% (Figure 4C). Information about the true index patient was not available [2]. However the spread was mainly nosocomial without any reports concerning the involvement of animals. The second NiV outbreak was reported in the district of Nadia, West Bengal in 2007 wherein all five NiV positive patients died within 10 days of infection leading to a fatality rate of 100% [40] (Figure 4C).

The third, recent, and most intense NiV outbreak occurred in the state of Kerala during May 2018, where 23 NiV positive patients were identified with a case-fatality rate of 91% [10,44]. The outbreak began on 2nd May, 2018 in Kozhikode with a 27 year old man who was admitted to hospital with a fever and myalgia. On the onset of high-grade fever, vomiting and altered sensorium, he was taken to another hospital but succumbed to death. His blood sample was not collected for testing the presence of NiV. The spread was recorded to be exclusively nosocomial; 22 cases contracted the NiV infection from the index patient. Out of the 23 cases, 2 survived and 21 died (18 affirmative NiV cases) with a highest mortality rate of 91% (Figure 4C) [10,45]. The outbreak was declared contained after 30th of May, 2018. Out of 18 clinical samples collected, all cases were verified positive for NiV RNA, NiV-specific IgM antibodies were detected in 13 patients whereas 4 patients were positive for IgG antibodies [10]. The index patient and sole infected person in the entire civic, was an ardent animal lover. He is assumed to have contracted the virus from an NiV-infected baby bat as the timing of the outbreak accords with the breeding season of bats. The transmission dynamics of the Kerala outbreak was mainly nosocomial [10]. Accounting for the total incidence cases in India, West Bengal outbreaks in 2001 and 2007 hold 70% and 5% of the total whereas Kerala reported 25% of all the Indian cases (Figure 4B). Outbreaks in both the states have shown alarming mortality rates.

In 2019, a recurring incidence of NiV was reported where one patient tested positive for NiV at the Ernakulam district of Kerala. A farsighted approach by the Kerala state government helped to tackle the 2019 outbreak. Approximately 300 contact persons, who had contact with the infected patient, were closely monitored for any possible Nipah-like symptoms. The index patient was moved to a strict isolation facility and keenly monitored, while the contact cases were advised to stay indoors and there was immediate reporting in case of any possible symptoms. As a precaution, monoclonal antibodies for the treatment of NiV were arranged from Australia in order to restrain any possible sporadic outbreak. Moreover, a fast and accurate diagnosis was facilitated by setting up test facilities within regional medical institutions. The recovery of the index case and containing the viral spread brought in huge accolades to the health sector and to government interventions in containing NiV during 2019.

#### 2.3.4. Clinical Features

During the Malaysian outbreak, the incubation period ranged from 4 days to 2 months, whereas in Bangladesh it was 10 days [40,46]. The incubation period ranged from 6–14 days in Kerala with a median of 9.5 days [10]. The major clinical presenting features of NiV include acute encephalitis with fever, head ache, vomiting and respiratory discomfort. Some patients also developed pneumonia, behavioral changes, disorientations with uncontrolled gait and low levels of consciousness [1,33,47]. During the Kerala outbreak, fever, myalgia, respiratory difficulties, headache, vomiting, cough, altered sensorium and encephalitis with seizures were reported in infected persons. Outbreaks in Bangladesh and India showed a higher number of cases with respiratory distresses [48].

### 2.4. NiV Strains Associated with the Outbreaks

Genomic sequencing have identified two strains of NiV: NiV-B and NiV-M. Genetic characterization of the two strains demonstrated that NiV-B has a genome size of 18,252 nucleotides which is longer than NiV-M by six nucleotides [49]. Even though both the strains share 91.8% similarity in nucleotide homology, NiV-B is considered to have higher fatality rates [49,50]. 

The two NiV strains are responsible for the outbreaks in various geographical areas. Bangladesh and Indian outbreaks have reported the source of the virus to be NiV-B while the Malaysian outbreaks were reported to be from NiV-M [49]. Clinical reports have uncovered several differences between NiV-M and NiV-B outbreaks. NiV-B has a shorter incubation period compared to NiV-M [40,46]. The majority of NiV-B cases had respiratory symptoms as well as lethal encephalitis while NiV-M predominantly caused encephalitis with few signs of respiratory diseases [1,2,51]. In addition, NiV-B infection resulted in a higher mortality rate compared to NiV-M. This difference could be attributed to inadequate regional medical care [32,52]. Nevertheless, it could also be linked to differences in the genetic variations and pathophysiology inherent to each strain.

Although NiV-B has been reported to cause repeated outbreaks, surprisingly, all in vitro and in vivo therapeutic research have utilized the NiV-M strain rather than the more prevalent NiV-B strain. Animal models of hamsters, ferrets and African green monkeys have been used to study the infection dynamics and disease patterns caused by both strains [32,53,54,55]. Comparing NiV-M and NiV-B infections in hamsters, NiV-M showed rapid disease pathogenesis and cytopathic effects whereas disease progression, immune response and viral replication was delayed in NiV-B infected animals. NiV-M was also found to be more pathogenic during in vivo infections of hamsters [53]. Moreover, researchers developed a foodborne transmission hamster model with the NiV-B strain which could successfully reproduce the outbreak scenario of Bangladesh [54]. However, both strains presented comparable respiratory tract lesions in hamsters, suggesting that there are no intrinsic alterations between both the strains reported during the human outbreaks [55]. Notably, these results showed an increased disease progression and fatality rates in NiV-M-infected models, contrasting to the NiV-B infections in human outbreaks. The ferret model revealed a similar pathological presentation and disease upon NiV-M and NiV-B infections. However, the NiV-M-infected ferrets’ blood measured a higher viral load while a higher rate of virus shredding was observed in the oral secretions of NiV-B-infected ferrets suggesting a valid reason for the high person-to-person transmission rates in NiV-B outbreaks [32], although hamster and ferret models developed disease on NiV infection but varied in pathophysiology from human outbreaks. 

NiV-M and NiV-B studies with African green monkeys demonstrated a 50% mortality on NiV-M infection while all experimental subjects infected with NiV-B succumbed to death [56]. Additionally, they could replicate an African green monkey model which demonstrated extremely severe respiratory distress and high fatality rates as observed in NIV-B-infected human outbreaks [56]. In spite of these experimental data, more research is required to elucidate the role of NiV genes and proteins in disease pathogenesis. The similarities in symptoms of NiV infection in *in vivo* African green monkeys and clinical presentation of NiV in humans may be attributed to the close relation of monkeys to humans in comparison to ferrets and hamsters.

### 2.5. Diagnosis

Early diagnosis is very critical for NiV infection as serious case fatalities are a hallmark of the disease. Various samples are collected from infected individuals and animals for diagnostic purposes. Specimens collected from humans include nasal swab, throat swab, urine, blood and cerebrospinal fluid (CSF) whereas lung, spleen, and kidneys from dead animals are used to diagnose and isolate NiV [57]. Diagnosis is performed in enhanced BSL3 (BSL3+) or BSL4 facilities. Diagnostic tests for detection of NiV include molecular and serological assays, immunohistochemistry, histopathology, virus isolation and neutralisation [58]. Vero cells are used to culture NiV with observable cytopathic effects in three days [57].

The most preferred and extremely sensitive diagnostic method is PCR. The conserved segments of viral genome, N, M and P are often targeted for the reverse-transcription PCR (RT-PCR) and nested PCR analysis. Though expensive, real-time RT-PCR, because of its extreme sensitivity, is employed extensively for NiV detection/diagnosis. Sensitivity of these techniques can be compromised if the viral genome undergoes rapid mutations [58,59].

Next generation sequencing is an alternative method aiding in effective identification of viral strain, however the method is not frequently used in diagnosis and when considering expenses. Another safe method for detecting NiV is immunohistochemistry since it uses formalin-fixed tissue samples [58,59]. ELISA is an effective serological assay which aids in the detection of NiV antigens and antibodies in serum samples. ELISA is often followed by a serum neutralisation test or PCR. Virus isolation and neutralisation methods are also used for diagnosis but are constrained to BSL-4 facilities [57,58,59].

### 2.6. Treatment

Rapid spread via nosocomial and zoonotic modes with an asymptomatic incubation period contribute to the potency of NiV outbreaks. Symptoms of NiV infection develop with instances of recurring mild fever progressing to a severe case of acute respiratory distress syndrome, acute encephalitis, altered sensorium and disorientation [60]. NiV infection needs high attention as there are no specific antivirals or antibodies presently effective against the infection. Supportive therapy with broad spectrum RNA virus antivirals like Ribavirin along with other medicines for deep vein thrombosis, anticonvulsive during seizures and mechanical ventilation on respiratory system failure are provided to rescue patients with NiV conditions [61]. 

Ribavirin and Acyclovir are two drugs used during the earlier outbreaks of NiV in Malaysia and Singapore [28,62,63]. Although Ribavirin reduced the death toll by 36% during the open label trial against Malaysian NiV outbreaks, subsequent studies in animal models failed to prove its efficacy [64]. Favipiravir (T-705), a purine analogue inhibiting RNA-dependent RNA polymerase progressed to clinical trials for Ebola and various types of influenza antivirals have also shown efficacy against NiV in Syrian hamster animal models [65,66]. 

Hendra virus subunit vaccine, an approved veterinary vaccine for equines in Australia, and a monoclonal antibody vaccine (m102.4) targeting the NiV recombinant viral envelope protein have proven their efficacy in various animal models; the latter has been administered on compassionate grounds to critical NiV patients [67]. Non-human primates (African green monkeys) challenged with NiV survived the infection on administration of the vaccine (m102.4) even after symptomatic conditions, providing hope for similar efforts looking towards successful medicine/vaccine development.

### 2.7. Prevention

Awareness among public and health care professionals towards neglected and similar diseases and a preparedness to contain any future outbreak by the medical and government authorities in regions with previous outbreak history should be ensured periodically. Studies towards understanding the bat environment, its susceptibility to being a carrier of NiV and its precise prevalence could help prevent the risk involved by human intervention into their habitat. Sampling and sero-surveillance for the NiV antibody and NiV with ELISA and PCR method in humans as well as bats will help to keep a check on the possibility of an outbreak in the prevalent regions [58]. 

Outbreaks similar to Kerala could be prevented by minimizing the patient-to-caretaker/attendant exposure via bodily fluids and close contact. Food materials, mats and sheets, dresses and patient aid materials of all sorts should only be handled when wearing masks and gloves. Proper incineration of the patient aids, the gloves and masks worn by the caretaker/attendant should be ensured. To reduce contracting the disease and its nosocomial spread, medical practitioners should wear proper personal protective equipment (PPE) while consulting encephalitic patients demonstrating NiV symptoms and should be moved to isolation from public on confirmation. Incineration of the corpse are the best practice, although burial of the corpse of the infected are not warranted, deep burial to a depth of 10ft along with proper PPE and disinfection of the handlers and burial land was followed in Kerala for particular cases on religious grounds. Contact with tissues or body fluids of the corpse may transmit NiV to healthy people [52]. Therefore, incineration or deep burial is necessary to minimize the risk of corpse-to-human transmission of the virus. However, it is unclear how long the infectious virus can survive in carcasses because no such data are available yet. Developing nations unable to afford the excessive demand for PPE’s should ensure that attendants and caretakers are cleaning their hands and body extremities with disinfectants like 70% alcohol or washing with soap and water. 

Procurement and consumption of raw fruits and products under unhygienic conditions in areas of NiV prevalence should be strictly prohibited. Bat-bitten fruits consumed raw are assumed to be one among the various reasons behind the recent outbreak in Kerala, very similar to the consumption of raw date palm sap contaminated with bat excreta leading to periodic outbreaks in Bangladesh [9]. Proper awareness and observation of hygienic methods could curtail the risk of acquiring and spreading diseases. Public broadcasting, flyers and posters as well as use of social media for proper awareness could be resorted to in foreseeing an outbreak period.

### 2.8. Control

Control at its best is the proper identification of the index case and further isolation containing the outbreak. A similar approach was made during the 2019 incidence of NiV in Kerala, India. A thorough study on the susceptibility (risk) and possible mode of spread in various regions could shed more light into the control measures to be observed. 

In Malaysia, on NiV identification, Japanese encephalitis-suspected pig farms known to harbor the NiV virus were isolated and classified as priority area 1; farmers and pig handlers were evacuated and culling of pigs in masses were done to reduce a further spillover of NiV [29]. A registry of the evacuated was prepared, screened for NiV and classified as encephalitis patients, community farm control and case controls. Broad spectrum antiviral therapy was followed to contain the outbreak. As a precautionary measure, all domestic transport and international export of pigs were restricted [29].

Persistent and periodic outbreaks have been reported from villages in Bangladesh from 2001. On identification of an outbreak cluster, villagers were made aware of the cause and were advocated against the raw consumption of the date palm sap. Consumption after boiling the molasses or the sap was advised along with a proper covering of the vessels used for sap collection [68]. Towards controlling the person-to-person spread, caretakers were made aware of the importance of cleaning hands after handling the infected patient and corpse with soap and water, which was mandated.

Though outbreaks have been reported in India coinciding with the first Bangladesh outbreak, in Siliguri and Nadia of West Bengal state, specific pathogens leading to the outbreak could not be identified. Misconception due to the prevalence of Japanese encephalitis in the region led to identification as an NiV outbreak. Kerala being naïve to NiV infections had no previous records of an acute encephalitic, severe respiratory distress syndrome. Similar to earlier outbreaks, the index cases were assumed to be infected with Japanese encephalitis and had been rationally diagnosed to be an NiV infection. Proper health measures with well-instructed protocols and high vigilant government support helped in systematically containing a highly lethal and sporadic nosocomial outbreak with very few victims. 

Regional administration and the state government with the support of the national government announced the scenario to be a national emergency situation. Proper awareness and strict protocols formed by the national health authorities to be observed towards handling the condition were publicized widely through printed media, public broadcasts and social media. High alerts were made in affected areas and public meetings, gatherings and the conduct of mass events were revoked to contain further spread through close aids. Strict measures against misleading messages were ensured. Records of the ill residents from the affected area were mandated towards proper surveillance of the outbreak. Rigorous sampling and testing for any new NiV infection incidences along with individuals having visited the affected area were monitored. All control measures were withdrawn upon proper assertion of zero incidence cases with double the incubation period. Towards reducing the disease burden, governments approached international agencies for the urgent supply of antivirals and vaccines under trials. A very similar approach towards controlling and containing an annual successive outbreak succeeded with a single infection and no casualties. Proper observation of the preventive measures with proper awareness among the public lead to the successful containment.

### 2.9. Factors Contributing to Outbreaks

#### 2.9.1. Population Density

Human population is on the rise and is always dependent on factors such as availability of resources, climate, trade and development. The presence of NiV has been reported in some of the most densely populous regions of the world, the South East Asia region (SEAR) [69]. The region represents only 5% of global landmass but contrastingly accounts for 26% of the world’s population. The world’s densest urban area happens to be in Bangladesh [70] whereas Kerala, the south Indian state, is considered among the most densely populated states in India. Population density also accounts for the high rate of interaction among individuals and between environments. Co-existence of farm animals in dense human inhabitation has a high risk of virus spillover. Populations of reservoir hosts, intermediate hosts and dead-end hosts may overlap in denser populations leading to a sporadic spillover and species barrier breach.

#### 2.9.2. Socio-Economic Scenario

Economy, poverty and population are defining factors of a nation’s strengths. A majority of countries in the SEAR are underdeveloped or classified as developing nations. Pandemics, epidemics and natural calamities agonize the lives of citizens in these nations. Although pig farming has been a major source of income for farmers, the NiV outbreak in Malaysia roots from pigs and pig sties [71]. Massive culling of pigs as a result of the outbreak lead to poverty and rehabilitation in the areas affected. Similarly, date palm sap consumers, which are major villages in Bangladesh have a high toll of NiV infection periodically [68]. West Bengal and Kerala are among the most literate and socio-economically developed states in India and have contradictorily been reported to have incidences of NiV. Transportation, tourism, high proportions of health care units in both the states accounts for the high rate of nosocomial NiV incidences.

#### 2.9.3. Deforestation and Climate Change

Deforestation in the SEA region is considered to be happening at an alarming rate due to gracing and farmland development, industrialization and urbanization. Deforestation has been linked to human interactions with Ebola-infected bats and the outbreak of Ebola in Africa [72]. Similar to the Ebola outbreak, deforestation has been understood to be the major cause of the 1998–1999 outbreak of NiV in Malaysia due to closer contact with NiV-infected bats [73]. The Malaysian NiV outbreak happened following the drought due to the El Nino [74]. Similar droughts resulting from El Nino have been reported from Kerala in 2016, though changes in the fruiting and pattern of bat foraging needs to be studied. Drought in the regions could reflect in a reduced availability of natural fruit forcing the bats to resort to fruiting trees in gardens and orchards existing in dense human habitats. Crop raising and fruit trees in close proximities to traditionally designed pig sties have contributed to the spillover of NiV to pigs via bat-bitten fruits [74]. 

#### 2.9.4. Intervention to Reservoir Habitats

Deforestation accounts for the major loss in bat habitats. Pteropus bats are frugivorous and nectarivorous and are known to reside in tropical forests across continents. They help disperse seeds of native and agro-economically important plants and crops. They are the sole pollinators in many oceanic islands apart from restoring the genetic diversity in intervened forest lands. Fruit collection, deforestation and tourism have contributed heavily to habitat loss. Human intervention in various modes reduce bat foraging, propensity and feeding habits and could impact on their physiology and immunology. Potentiating the hypothetical ideas on viral dynamics among the bat populations and their offspring, horizontal in contrast to vertical transmission has been identified as a major mode of NiV spread among the bat reservoir population [75].

#### 2.9.5. Reservoir Distribution (Demography)

Chiroptera and Pteropodidae are bats known to be old world fruit bats and are critical for seed dispersal and pollination in tropical and paleotropic forests in Asia, Africa, Australia and many tropical regions around the globe. They help improve the plant diversity among various forest vegetation along with pollination and the spread of rare plant species native to the region. Habitat loss has forced many populations of bats to remain in urbanized parts of the world, constantly foraging in farms and orchards in close vicinity of human settlements [76,77].

#### 2.9.6. Virus Shedding and Stress in Reservoir Hosts

Factors leading to virus shedding include physiological and environmental bat stress, weakened immunity, reduced maternal immunity, infection in new born and roost population and interaction, ultimately reducing food availability [75]. Hypotheses with broader and differing concepts have been employed for a better understanding of the phenomena. Spillover and different episodes of pathogen shedding has been conceptualized from the persistent infections existing within bat populations during weakened immunity. High antibody titer and persistent pathogens from healthy bats form the base to the concept.

Transient epidemic waves are short pulses of pathogens re-establishing in roosts or new pathogens colonizing due to resident pathogen clear off among metapopulations of bats. Viruses of low virulence could infect and spread in and out of populations suppressed by the bats immunity and could re-emerge on immunity reduction along with other stressing factors leading to a short pulse or waves of infection [78]. Bats persisting in urban areas and their migratory patterns, population size, lifespan and immune status can contribute to spillover and spread [77]. Additionally, the gregarious nature of bats among populations and its environment promotes its status to a supercarrier.

## 3. Conclusions

Nipah viral outbreaks documented in various parts of the world pose a substantial threat to the global community. Economies of the affected countries have been struck severely by these outbreaks, grounding to a high rate of morbidity and mortality and eventually declining their economic stability. Preparedness and proper awareness among populations could benefit in controlling and containing outbreaks of any dimension. Enumerating the factors influencing the outbreak, surveys and studies to better understand the virus dynamics among inter species populations with preventive measures could curtail future outbreaks to a greater extent. Authorities and governments should practice and follow preventive and containment measures towards controlling any possible sporadic outbreak of similar nature.
